# A comparative study using response surface methodology and artificial neural network towards optimized production of melanin by *Aureobasidium pullulans* AKW

**DOI:** 10.1038/s41598-023-40549-z

**Published:** 2023-08-19

**Authors:** WesamEldin I. A. Saber, Abeer A. Ghoniem, Fatimah O. Al-Otibi, Mohammed S. El-Hersh, Noha M. Eldadamony, Farid Menaa, Khaled M. Elattar

**Affiliations:** 1 Microbial Activity Unit, Department of Microbiology, Soils, Water and Environment Research Institute, Agricultural Research Center, Giza, 12619 Egypt; 2 Botany and Microbiology Department, Faculty of Science, King Saud University, 11451 Riyadh, Saudi Arabia; 3 Seed Pathology Department, Plant Pathology Research Institute, Agricultural Research Center, Giza, 12619 Egypt; 4 Department of Biomedical and Environmental Engineering (BEE), Fluorotronics, Inc. California Innovation Corporation, San Diego, CA 92037 USA; 5 Unit of Genetic Engineering and Biotechnology, Faculty of Science, Mansoura University, El-Gomhoria Street, Mansoura, 35516 Egypt

**Keywords:** Biotechnology, Microbiology

## Abstract

The effect of three independent variables (i.e., tyrosine, sucrose, and incubation time) on melanin production by *Aureobasidium pullulans* AKW was unraveled by two distinctive approaches: response surface methodology (i.e. Box Behnken design (BBD)) and artificial neural network (ANN) in this study for the first time ever using a simple medium. Regarding BBD, sucrose and incubation intervals did impose a significant influence on the output (melanin levels), however, tyrosine did not. The validation process exhibited a high consistency of BBD and ANN paradigms with the experimental melanin production. Concerning ANN, the predicted values of melanin were highly comparable to the experimental values, with minor errors competing with BBD. Highly comparable experimental values of melanin were achieved upon using BBD (9.295 ± 0.556 g/L) and ANN (10.192 ± 0.782 g/L). ANN accurately predicted melanin production and showed more improvement in melanin production by about 9.7% higher than BBD. The purified melanin structure was verified by scanning electron microscopy (SEM), energy-dispersive X-ray spectroscopy (EDX), X-ray diffraction pattern (XRD), and thermogravimetric analysis (TGA). The results verified the hierarchical architecture of the particles as small compasses by SEM analysis, inter-layer spacing in the XRD analysis, maximal atomic % for carbon, and oxygen atoms in the EDX analysis, and the great thermal stability in the TGA analysis of the purified melanin. Interestingly, the current novel endophytic strain was tyrosine-independent, and the uniquely applied ANN paradigm was more efficient in modeling the melanin production with appreciate amount on a simple medium in a relatively short time (168 h), suggesting additional optimization studies for further maximization of melanin production.

## Introduction

The curiosity towards natural pigments, especially those from microorganisms, has attracted consumers, since the widespread of synthetic dyes in cosmetics, food processing, textile, and pharmaceuticals became limited, owing to their carcinogenicity, hyper allergenicity, and toxigenicity^1,2^. The value of microbial pigments includes their high stability, yield, and easy production, as well as their low cost and ease of microbial cultivation^3^. Microbial melanin production is a talented field of investigation, due to the growth of the industry demanding natural pigments as safe products, effortlessly degradable and eco-friendly^1^.

Melanin is a dark-brown pigment that is formed by the oxidative polymerization of phenolic compounds, such as glutaminyl-3,4-dihydroxybenzene, catechol, 3,4-dihydroxynaphthalene, or 3,4-dihydroxy-phenylalanine, during the metabolic pathways of fungi, another route is the 3,4-dihydroxyphenylalanine pathway, where tyrosine is catalyzed by tyrosinase and laccase to form dopaquinone, which is oxidized and auto polymerized to melanin^3,4^. Additionally, melanin production depends upon intra and/or extracellular tyrosinase enzymes, by which several studies attained the kinetic process of activation, inactivation, inducer, and inhibitors of tyrosinase enzyme during growth of *Penicillium chrysogenium, Trichderma reesei* and *Trichoderma harzianum*^2,5^.

Among microbial melanin producers, fungi have a superiority in melanin construction with availability as a potent source, because they are proficient in producing a high yield of the substance in the low-cost culture medium, which crafting the bioprocess to economically practicable on the industrial scale^3,4^. Several ascomycetous fungi were reported to produce melanin *e.g*., *Aureobasidium pullulans*, *Neophaeotheca triangularis*, *Trimmatostroma salinum*, and *Hortaea werneckii*^6,7^. In comparison to bacteria, these fungi have potential advantages due to the predominancy of two key enzymes, (tyrosinase, and laccase) that play a major role in melanin production^8^.

Another virgin area of microorganisms had to be investigated and explored for melanin production i.e., the endophytic microbes, which are more efficient in the production of unique biomolecules in appreciated amounts. Endophytes refer to microbial communities that live and grow within plant tissues without causing any harm to the host^9^.

Melanin has several feasible applications in the medicinal field e.g., antimicrobial, antiviral, antioxidant, antitumor, and anti-inflammatory, as well as, in cosmetics as a protective agent for the skin and eye^3,10^, and environmental bioremediation of polluted sites by heavy metals^11^. Owing to these melanin benefits in a variety of applications, there is a growing request for melanin production in a low cost-medium. The bioproduction process could be substantially reduced using a new optimization concept^12^.

Response surface methodology (RSM) is a multivariate statistical design applied for evaluating the effects of factors, constructing models, and predicting optimal conditions with the minimum number of runs^9,13,14^. RSM is a statistical technique that models the correlation between multiple independent variables and the response of bioprocess. It is commonly applied to optimize bioprocess performance by identifying the optimal settings for a set of variables that influence the response. RSM is an efficient way for the design and analysis of experiments to optimize bioprocess performance. This, in turn, allows the determination of a strong spot where the parameters can achieve the highest yield and the lowest possible operation parameters, and error^9,13^.

Central composite designs (CCD), and BBD are both RSM that can be used to fit a full quadratic model. BBD was selected because it does not include axial points that are located outside the cube of the design space to estimate the curvature of the response surface; thus, BBD has fewer design points and saves time and money, compared to that of CCD. These points are not necessary if the region of interest is well-behaved, as it is the case in our study here. For the same reason, BBD is safe to run, because there are no extreme runs, and the factors performed well at their tested limits. Because of the rotatable nature of BBD, it is more robust to noise, meaning that not sensitive to the order of the experimental runs, i.e., does not need to run the design in a specific order^15–17^.

BBD involves varying the input variables (factors) within certain predetermined ranges and levels, allowing for efficient use of resources and reduction in experimental runs^18^. The design can also be useful for studying the interactions between variables and identifying the optimal combination of variables that maximizes the response variable^19^. The review of literature contains a plethora of reports highlighting the potential of RSM, in particular, the BBD, in the optimization of numerous bioproducts like enzymes (α-amylase^15^, β-xylanase^20^, pectic oligosaccharides production^21^, biodegradation of antibiotics in the environment^17^, and monascus pigment production^16^, etc.

No previous work was carried out on the optimization of melanin production using artificial intelligence. Artificial neural network (ANN) serves as a core element and one of the basic tools utilized in machine learning. ANN could be considered an advanced extension of RSM and could terrifically, substitute the polynomial regression-based modeling approach of RSM^22–24^. Like the human brain, an ANN is capable of sophistically analyzing and processing data by efficiently constructing computational models with fully connected nodes within the hidden layer(s). This type of modeling facilitates the learning of data patterns and, as a result, enables accurate decision-making based on historical data. When constructing an ANN model, the network architecture is first selected, followed by the creation of hidden layer(s) with sufficient neurons. The network then undergoes a learning and training process until it grasps the data pattern. Once completed, the resulting ANN model is validated and verified before it is approved as a predictive model. The paradigm of ANN is based on identifying various patterns in the data and detecting any differences to determine the pattern that achieves the desired outcome. This process is regulated through intelligent backpropagation, which generates the desired output model to achieve the objective (target). This procedure is more accurate and can effectively replace other modeling methods^22,25^.

In this study we aimed to develop a simple medium that contains the minimal requirements for melanin production (i.e., tyrosine, and sucrose, in addition to the incubation period). The reason behind the selection of tyrosine is that the biosynthesis of melanin depends upon tyrosine as the precursor for melanin production^3^. Many sources of carbon could be metabolized by fungi, but no documented data indicated the role of sucrose or carbon sources in melanin synthesis by fungi, but they can be involved in various metabolic processes by most fungi for energy and growth^26^. Herein sucrose was selected as a readily available and cheap carbon source. Fermentation time is crucial for melanin production as it determines the duration and extent of melanin synthesis.

However, although the commercial production of melanin from microbial origin has seen significant advancements^27^, there is still room for improvement. Therefore, our study aimed to optimize melanin production using a combination of BBD and ANN methodologies. To the best of our knowledge, this is the first study to utilize ANN procedure to enhance melanin production from the endophytic fungus, *A. pullulans*, while utilizing an economical medium source.

## Materials and methods

### Fungus and inoculum preparation

*Aureobasidium pullulans* strain AKW was isolated and identified in our previous study^28^. The stock culture was preserved on potato dextrose agar plates after being incubated at 30 °C for 5 days under dark conditions, and sub-cultured periodically. For the preparation of the standard inoculum, *A. pullulans* AKW was grown in the same fermentation medium under shaking (100 rpm, 30 °C, 72 h) to obtain a cell count of approximately 3 × 10^7^ cells/mL.

### Core melanin production medium

For the melanin production process, a simple fermentation medium *i.e*., potato sucrose broth was used. The medium was prepared using potato infusion as a base for the fermentation medium. To prepare potato infusion, 200 g of sliced, and unpeeled potatoes were boiled in one-liter distilled water for 30 min, then filtered to remove debris of potato residuals. Different concentrations of tyrosine (as an inducer of melanin production) and sucrose (as a carbon source) were added to the potato infusion (Table 1). The pH of the medium was adjusted to 6.0. Portions of 100 mL medium were transferred into canonical flasks and autoclaved at 121 °C for 15 min. The medium was inoculated by fungal inoculum in a proportion of 5%. The inoculated culture medium was incubated at 30 °C for different periods with a rotation speed of 200 rpm.

**Table 1:** Three-factors matrix used for melanin production using *A. pullulans* AKW, showing the actual and the predicted values of each point of Box-Behnken and ANN models.

Run		Independent variable			Melanin (g/L)				
					Actual ± SD	Box-Behnken		ANN	
No	Pattern	Tyrosine (%, w/v)	Sucrose (%, w/v)	Time (h)		Predicted	Error	Predicted	Error
1*	− − 0**	0.1	3	168	5.871 ± 0.490	5.695	0.176	5.857	0.014
2*	− + 0	0.1	7	168	6.954 ± 0.426	6.983	− 0.029	6.984	− 0.030
3*	+ − 0	0.5	3	168	5.529 ± 0.152	5.501	0.029	5.494	0.035
4	+ + 0	0.5	7	168	6.783 ± 0.227	6.959	− 0.176	6.959	− 0.176
5	0 − −	0.3	3	120	8.436 ± 0.325	8.505	− 0.069	8.375	0.061
6*	0 − +	0.3	3	216	3. 743 ± 0.255	3.878	− 0.135	3.749	− 0.006
7*	0 + −	0.3	7	120	8.341 ± 0.321	8.206	0.135	8.221	0.120
8	0 + +	0.3	7	216	6.992 ± 0.673	6.923	0.069	7.074	− 0.082
9*	− 0 −	0.1	5	120	7.904 ± 0.734	8.011	− 0.107	7.967	− 0.063
10*	+ 0 −	0.5	5	120	8.075 ± 0.523	8.035	0.040	8.167	− 0.092
11	− 0 +	0.1	5	216	5.149 ± 0.645	5.189	− 0.040	5.134	0.015
12	+ 0 +	0.5	5	216	5.054 ± 0.747	4.947	0.107	4.955	0.099
13*	000	0.3	5	168	9.804 ± 0.850	10.184	− 0.380	10.177	− 0.373
14*	000	0.3	5	168	10.811 ± 0.539	10.184	0.627	10.177	0.634
15*	000	0.3	5	168	9.973 ± 0.412	10.184	− 0.247	10.177	− 0.240

*Ten runs were used for the training process, whereas the other 5 runs were used for the validation process.

**The pattern represents the level composition of each variable within each of the tested runs, where + is the high level, − is the low level, and 0 is the middle concentration.

### Separation and purification of melanin

The protocol of El-Gamal et al.^29^ and Müjdeci^30^ were used for the separation and purification of melanin with minor modifications. Briefly, melanin was separated from the microbial cell pellets by centrifugation for 15 min at 3000×*g*. The resulting cell-free filtrate containing melanin was precipitated by reducing the pH down to 2.0 by HCl (6 M) and kept for 4 h under cooling. The precipitate was centrifuged at 7000×*g* for 15 min. The precipitated melanin was washed with distilled water. The process was repeated four times to obtain purified melanin. The purified pigment was stored (at − 20 °C) after lyophilization.

### Design of Box-Behnken matrix

This investigation aimed to study the best combination of the most important factors affecting melanin production on a simple medium. The study hypotheses that tyrosine (the precursor for melanin production), sucrose (the most common carbon source), and incubation time are significant factors. The three independent variables were optimized for maximization of melanin production using the BBD of RSM^31^. Based on the matrix of BBD, the independent factors were tested at three levels (low, middle, or high). Accordingly, 15 runs were generated that contain three center points (middle levels). Table 1 displays the experimental design matrix, which includes the various combinations of the three variables. Each combination was repeated three times. Upon performing the experimental trials as previously stated, melanin production was measured as the target response to the three-factors matrix. Then, the collected data were statistically analyzed to find out the relationship between the three independent variables and melanin production to predict the optimum level of each factor, the next Eq. (1) was applied:

1 $${{Y=\beta }_{0}+\sum \beta }_{i}{X}_{i}+\sum {\beta }_{ij}{X}_{i}{X}_{j}+{\beta }_{ii}{X}_{i}^{2}$$

where Y is the forecasted melanin production, *β*_*0*_ is the model constant, *β*_*i*_ is the linear coefficient, *β*_*ij*_ is the cross-product coefficient, *β*_*ii*_ is the quadratic coefficient, and *X*_*i*_*,* and *X*_j_ are the independent variables.

The equation was used to find out the connection between independent variables and melanin production, as well as the prediction of the optimum concentration of each factor. The analysis of variance (ANOVA) and coefficient of determination (R^2^) were performed to determine the significance of variables and ensure the goodness of the model. Moreover, the BBD precision was visually assessed by plotting the predicted values against the actual values and examining the distribution of the data points.

### Modeling melanin production with ANN

The obtained data from the BBD matrix was utilized for developing a predictive model with machine learning. The ANN model was created by training a fully connected ANN with two hidden layers, in which all nodes were equipped with a hyperbolic tangent sigmoid; exp(-x^2^) activation function. The established forecast model was a fully connected algorithm of multilayer perceptron.

Accordingly, the data were split into (i) the training set that was used to reduce the error and establish weights at each neuron of the ANN, (ii) the validation set that was used to tune hyperparameters and monitor model performance during training to halt the ANN training and select the best model, and finally (iii) the testing set that was used to evaluate the final trained model, as an external dataset that was solely used to test the ANN robustness and serve as the final valuation of prediction capacities; this external dataset was not used during the model development process^32^.

The ANN topology was investigated utilizing several hidden layers and nodes. The ANN topology was designated as 3-h-1. Three neurons of the tested factors (i.e., tyrosine, sucrose, and incubation time), represent the input layer. The output layer is composed of one neuron (fungal melanin production). Additional hidden layers were created between the two input and output layers, in which a range of neurons from 3 to 10 was tested.

ANN was trained using trial-and-error at various specific parameters (learning rate, learning method, and the ideal neurons number in the hidden layer). For each trial phase, a 10,000 tour was used. Then, the optimum architectural structure that fits the best model was identified. The trial-and-error process was employed untill the lowest error was for the root average squared error (RASE) and average absolute error (AAE). The coefficient of variation (R^2^) between the predicted and actual values was also used to evaluate the training process.

The precision of the trained ANN was determined by comparing its predicted outputs to the actual values of melanin production. If the predicted values were close to or equivalent the actual values, then the ANN was deemed to have high precision in its predictions. This evaluation was usually done by calculating a statistical measure of accuracy, RASE, AAE, and R^2^. Additionally, the ANN's precision was visually assessed by plotting the predicted values against the actual values and examining the distribution of the data points^24^.

### Verification

The optimum level of each of the tested factors that maximize melanin production was theoretically estimated using the fitted model of both BBD and ANN, then the expected melanin production was estimated. A verification trial was performed, applying the optimum settings in the laboratory in triplicates and compared with the predicted melanin values to test the accuracy of both models.

### Software and statistical analysis

Data of melanin production were presented as the mean ± standard deviation. The design of the BBD matrix and the statistical analysis were performed using the JMP® Pro 17 software (JMP®, SAS Institute Inc., Cary, NC). The software was also utilized for conducting machine learning procedures, constructing the ANN topology, and implementing the training, validation, and testing procedures.

### Characterization methods

The particles’ surface morphology of the purified melanin pigment was inspected by scanning electron microscope (SEM)^33^. The gold-coated melanin that had been previously lyophilized was examined using SEM-type FEI Czech (accelerating voltage at 25 kV), which consists of an electron optical column, vacuum system, and electronics. The electromagnetic lenses focused the electrons into a fine spot onto melanin. The electron gun, tungsten filament at the top of the column produced the electron beam, which is focused into a fine spot less than 4 nm in diameter on the melanin specimen. This beam is scanned in rectangular rashers. The compositional pattern was determined by energy-dispersive X-ray (EDX) spectroscopy^33^, which is connected to SEM.

Thermogravimetric analysis (TGA) was run on Perkin Elmer 4000^33^. The X-Ray diffraction pattern analysis (XRD) was run at Pan Analytical Philips according to Khouqeer et al.^34^. The Bragg equation (Eq. 2) was applied to calculate the inter-layer spacing *d*. The average grain (crystalline) size of melanin was estimated by the Dedye-Schrerrer equation (Eq. 3). The percentage of the melanin crystallinity was correspondingly calculated as stated in (Eq. 4), considering glass substrate as a background.

2 $$2d\,\mathrm{sin}\,\theta =\mathrm{m\lambda }$$

where, *θ* ≡ diffraction angle; m ≡ diffraction order; λ ≡ X-ray wavelength at the considered first-order diffraction (m = 1).

3 $$\mathrm{D}=0.9 \,(\uplambda /\mathrm{ FWHM}.\,\mathrm{cos}\,\theta )$$

where, λ denotes the wavelength (1.54) and FWHM ≡ full width at half maximum of a diffraction peak.

4 $$\mathrm{Percentage\,of\,crystallinity}=(\mathrm{Total\,background\,profile\,area}/\mathrm{Total\,area})\mathrm{ X }100$$

## Results

A simple medium of the yeast-like fungus, *A. pullulans* AKW, was sequentially optimized for melanin production via BBD and ANN paradigms. The resulting melanin was characterized based on SEM, XRD, EDX, and TGA properties.

### Modeling melanin production by BBD

Data from Table 1 displays the pattern of a 15-run design matrix of the different combinations of the three variables together with the experimental melanin production. Data obtained revealed a substantial variation in melanin production with the best levels combination at the center point (middle level), being 5.69 ± 0.39 (run number 14).

#### The statistical weight of the tested variables

**Figure 1:**
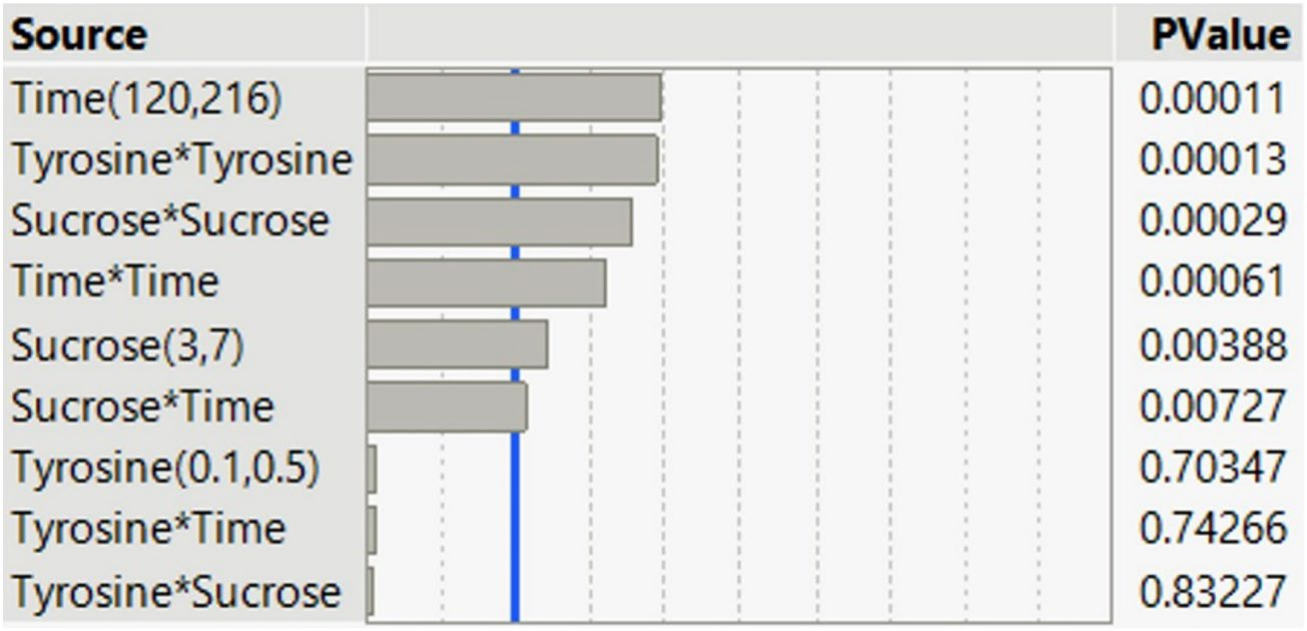
The relative single, interaction, and quadratic importance of the tested factors on melanin production by *A. pullulans* AKW.

The relative single, interaction and quadratic importance of the tested factors were quantified using the P-value for comparison. The statistical weight (Fig. 1) of the tested variables was used to detect which predictor (variable) is the most significant. The most important variable has the lowest *P*-value. Likewise, the single effect of incubation time and sucrose was significant, whereas tyrosine level did not show any significant influence on melanin production. Regarding the interaction effect, the interaction between tyrosine with the other two factors (incubation time and sucrose level) showed a non-significant effect. Eventually, the three variables showed a significant quadratic effect.

#### ANOVA and regression analysis

The data of BBD were analyzed by ANOVA (Table 2). The overall model is significant (*F-*value = 42.4805) with a very low *P-*value (0.0003), on the other side, the lack of fit error is not statistically significant (*P*-value = 0.9206). Other model evaluation statistics were additionally estimated i.e., the R^2^, adjusted-R^2^, and pridected-R^2^ values, which were found to be extremely high, being 0.9871, 0.9639, and 0.9382, respectively.

**Table 2:** ANOVA analysis of Box-Behnken design for melanin production by *A. pullulans* AKW.

Source	Degree of freedom	Sum of Squares	F-ratio	Prob > F
Overall model	9	56.139	42.481	0.001
Error				
Lack of fit	3	0.136	0.151	0.9206
Pure error	2	0.599		
Total error	5	0.734		
Single				
Tyrosine (0.1, 0.5%, w/v)	1	0.024	0.163	0.7035
Sucrose (3, 7%, w/v)	1	3.769	25.667	0.0039
Time (120, 216 h)	1	17.458	118.895	0.0001
Interaction				
Tyrosine*Sucrose	1	0.007	0.050	0.8323
Tyrosine*Time	1	0.018	0.121	0.7427
Sucrose*Time	1	2.796	19.039	0.0073
Quadratic				
Tyrosine*Tyrosine	1	16.534	112.602	0.0001
Sucrose*Sucrose	1	11.746	79.996	0.0003
Time*Time	1	8.557	58.278	0.0006
Corrected total	14	56.873528		

Model evaluation statistics				
Determination coefficient (R^2^)	0.9871			
Adjusted-R^2^	0.9639			
Pridected-R^2^	0.9382			

The single, interaction, and quadratic terms were also investigated by ANOVA. Apart from tyrosine, sucrose and incubation time were significant. Concerning the interaction among variables, the interaction between sucrose level and incubation time has a significant effect (*P*-value = 0.0073). Otherwise, all the quadratic terms showed a significant effect (*P*-value ≤ 0.05) on melanin production mediated by *A. pullulans* AKW.

Consequently, the regression coefficients of the various terms of the model were computed and obeyed to a polynomial function of the second order. The predicted melanin production by *A. pullulans* AKW was estimated by the next equation:

$$\begin{gathered} Y = 10.184 - 0.055\left( {{\text{X}}1} \right) + 0.686\left( {{\text{X}}2} \right) - 1.477\left( {{\text{X}}3} \right) \hfill \\ + 0.043\left( {{\text{X}}1{\text{X}}2} \right){ } - 0.067\left( {{\text{X}}1{\text{X}}3} \right) + 0.836\left( {{\text{X}}2{\text{X}}3} \right){ } \hfill \\ - 2.116\left( {{\text{X}}1} \right)^{2} - 1.784\left( {{\text{X}}2} \right)^{2} - 1.522\left( {{\text{X}}3} \right)^{2} \hfill \\ \end{gathered}$$

where Y is the predicted melanin production, X1 is tyrosine (%, w/v), X2; is sucrose (%, w/v), and X3 is the incubation time (h).

As could be noticed, some model terms (X1, X3, X1X3, and all quadratic terms) showed negative coefficient values, whereas the others had positive coefficient values. However, such an equation was used for the prediction of melanin production values, which were found to be very close to the experimental ones and therefore had lower errors. Based on the above regression model, the values predicted by BBD were estimated and compared with actual values. Both estimated and actual values at each data point exhibited high agreement (Table 2).

### Modeling melanin production by ANN

**Figure 2:**
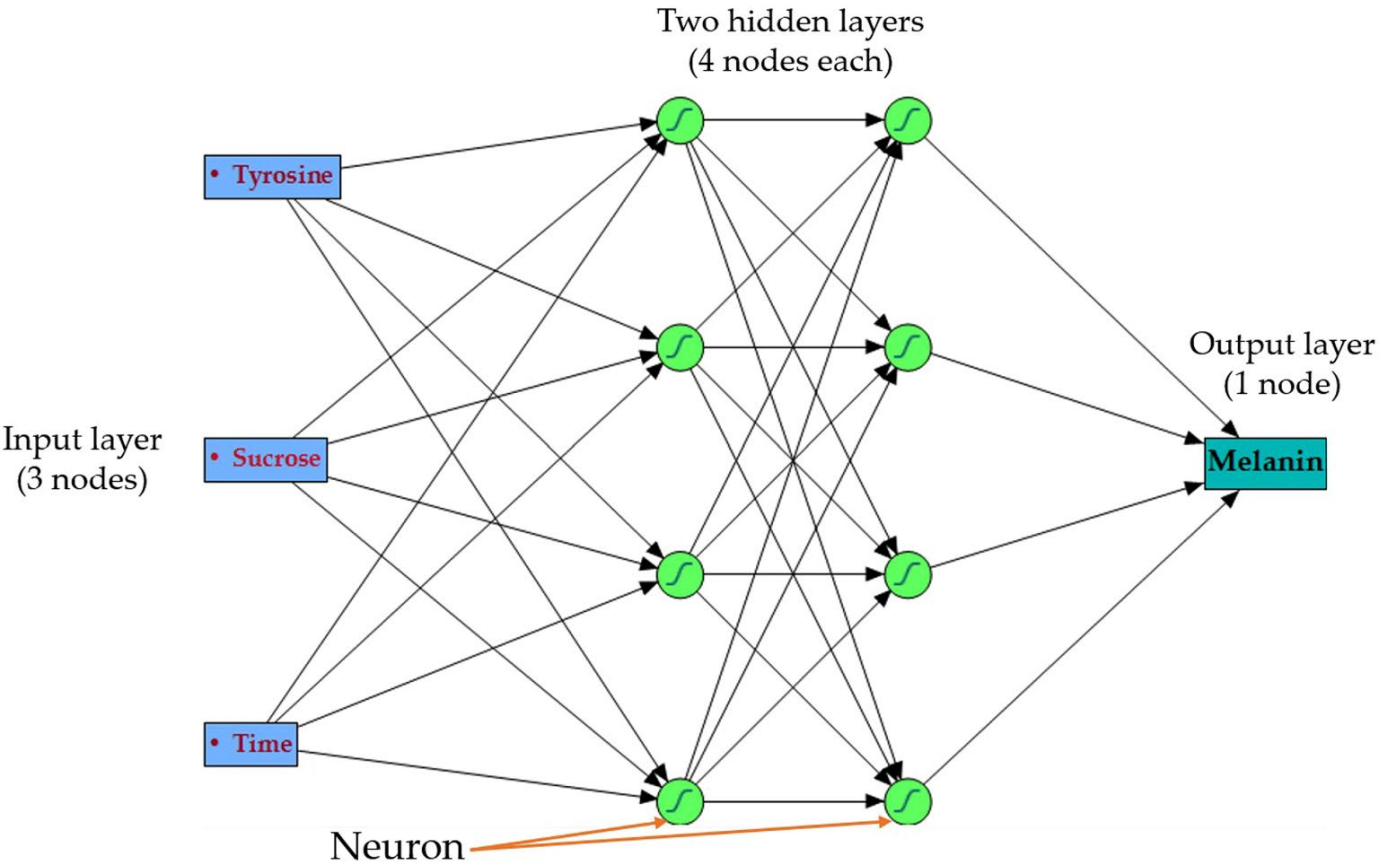
The ANN layout of melanin production by *A. pullulans* AKW. The ANN contains an input layer (three neurons), two hidden layers (4 neurons, each), and the output layer (one neuron).

A multilayer feed-forward ANN design was employed in a neural network platform that is fully connected to construct a predictive ANN model. During the trial-and-error procedure, the holdback propagation ratio was used at 0.3333, which divided the data of BBD into two sets, the first set having 10 runs for training to reduce, prediction error and create neural weights. The second set is composed of 5 runs for validation to stop ANN training and choose the finest model. After several trials (each of 10,000 tours) with various ANN arrangements. The penalty of ANN topology was best achieved at a squared learning rate of 0.2, with two hidden layers each with 4 nodes. The activation function in all nodes of both hidden layers of the network was hyperbolic tangent sigmoid (NTanH). As a result, a four-layer ANN topology structure (Fig. 2), denoted as 3–4–4–1, was determined to be the optimal architecture. The input layer consists of three neurons (independent factors), which are tyrosine, sucrose, and incubation time. The neuron-output layer represents the response factor of melanin production. The two hidden layers demonstrated superior performance when employing the activation function of NTanH(4)NTanH2(4) in the hidden neurons.

To test the generality of the model, trials were made to minimize errors during the machine-learning process. The ANN was trained until the R^2^ value was maximized, achieving a score of 0.9857 for training, and 0.9935 for validation.

The trained network's ability to accurately predict outputs was evaluated. The predicted values for each point of the BBD data were computed using the ANN (Table 1). The values predicted by ANN exhibited high consistency with the investigational values and displayed lower error values than those of the BBD model.

### Assessment of BBD and ANN models

#### Residual analysis, and linear relationships

**Figure 3:**
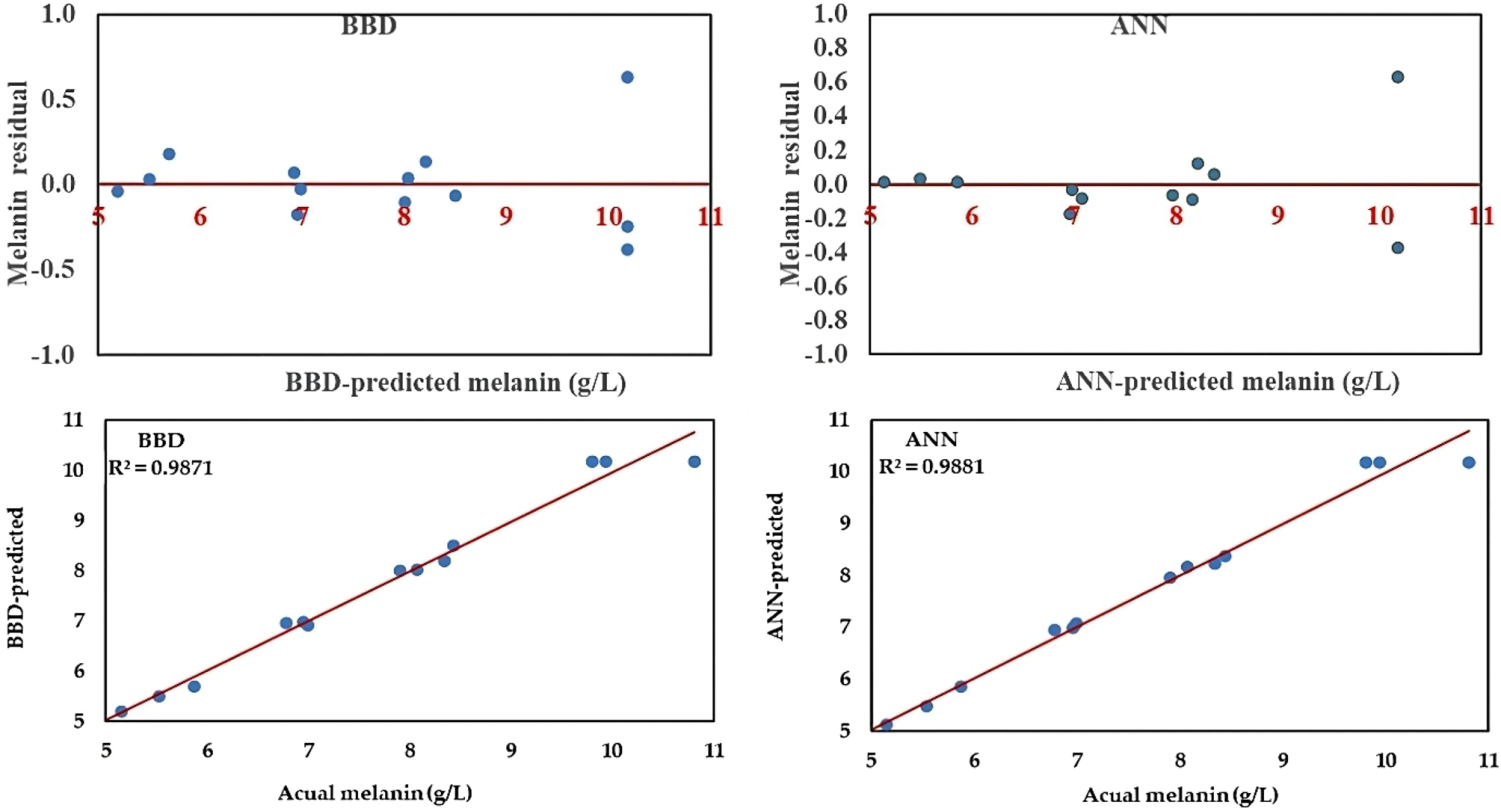
Plots of the BBD and ANN, showing the predicted values of melanin production versus residual, and actual data.

Residual analysis was performed for further assessment of both models to predict melanin production by *A. pullulans* AKW. The plotted residuals versus predicted values by both models (Fig. 3) revealed an equal distribution of the residual points that were scattered closely, and equally around the 0-axis without exhibiting linearity.

The predicted model points against the actual experimental values of both models (Fig. 3) were also investigated. The points of both models lie near the line of perfect prediction, implying that the model can approach the actual experimental data faithfully. However, the linear regression showed that the ANN model's predictions are significantly closer to the prediction line compared to the BBD model.

#### Three-dimensional (3D) plots

**Figure 4:**
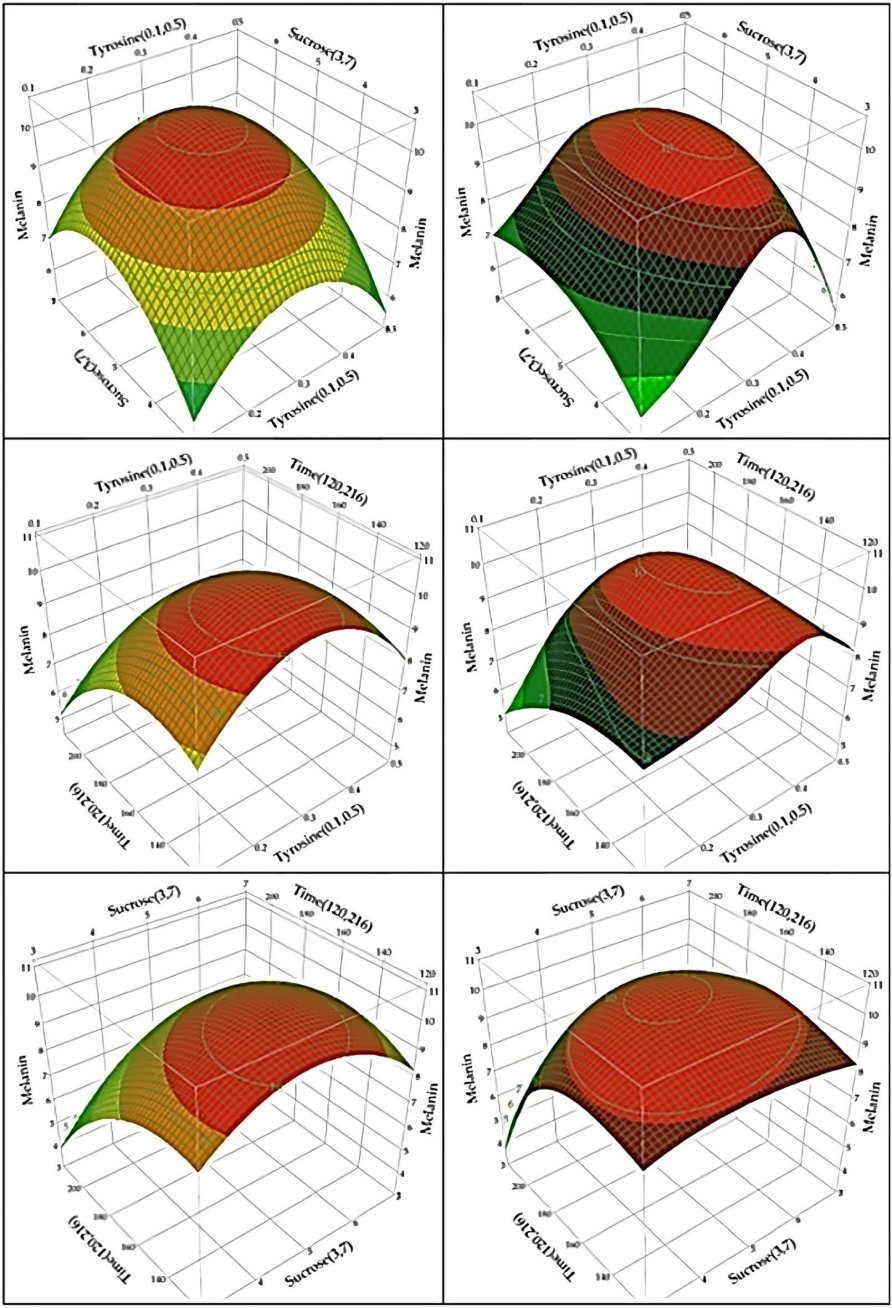
Three-dimensional response surface plots of melanin production by *A. pullulans* AKW, represented as a pairwise combination of the tested factors, keeping the third-factor constant at its center (middle) level, based on the models of BBD (represented by the right column) and ANN (represented by the left column).

To know the mutual, and interactive effects between every pair of variables, the 3D plots of both BBD and ANN were constructed (Fig. 4). The 3D plots depicted the relationships between the variables, demonstrating an increase in melanin production as the concentration of the three variables (i.e., tyrosine, sucrose, and incubation time) approached the optimum level. However, once the optimum level was exceeded, a decline in melanin production was observed. It can be noticed that higher levels of the three variables have a negative effect on the response.

Compared with BBD, ANN demonstrates an elliptical curve of the response surface plot between each pair of the tested factors. The 3D plots of BBD showed various patterns, and no specific prototype could be drawn for every pair of the tested factors, where the elliptical curve appeared only on the interaction between tyrosine and sucrose levels. In general, the 3D plot indicates that the melanin production process reached its maximum value around the central points of the experimental design.

### Comparison of the models

Both the BBD and ANN models exhibited strong predictive ability and lower residuals, indicating that they can accurately fit actual investigational data and make highly reliable predictions. To evaluate the precision of these models in predicting melanin production, various statistical parameters were assessed during both the training and validation processes, as well as for the overall models (Table 3). Based on the performance statistics, the ANN model was more confident than the BBD model, where the R^2^ value of the overall ANN model was higher than these of the BBD model. On the other hand, RASE and AAE values recorded lower values for the ANN model than for the BBD model.

**Table 3:** Measuring the performance statistics, prediction, and the actual response of BBD and ANN models for melanin production by *A. pullulans* AKW.

Model		R^2^	RASE	AAE	Frequency
Training	BBD	0.9845	0.2609	0.1905	10
	ANN	0.9857	0.2506	0.1607	10
Validation	BBD	0.9933	0.1034	0.0921	5
	ANN	0.9935	0.1016	0.0868	5
Overall	BBD	0.9871	0.2212	0.1577	15
	ANN	0.9881	0.2128	0.1360	15

Variable	Predicted value				
	BBD	ANN			
Predicted optimum conditions					
Tyrosine (%)	0.299	0.329			
Sucrose (%)	5.168	4.870			
Incubation time (h)	145.825	167.994			
Validation of response					
Predicted melanin production (g/L)	10.554	10.286			
Experimental melanin production (g/L)	9.295 ± 0.556	10.192 ± 0.782			

*RASE* root average squared error, *AAE* average absolute error.

### Model verification

A verification experiment was conducted based on the predicted values of each factor by both models, to assess the modeling process used in the optimization study of melanin production. The predicted optimal conditions were calculated separately by BBD and ANN models (Table 3). Then the calculated values of the three tested factors were experimentally validated. Under the theoretical optimum conditions for the three variables, the response of melanin production was calculated to be 10.555 and 10.286 g/L for BBD and ANN, respectively. The validation process revealed high consistency with the experimental melanin production, being 9.295 ± 0.556 g/L (BBD) and 10.192 ± 0.782 g/L (ANN). It is worth mentioning that both models showed high accuracy, but ANN surpassed the BBD model in predicting melanin production, achieving a 9.7% increase in melanin production over BBD.

### Characterization of melanin

**Figure 5:**
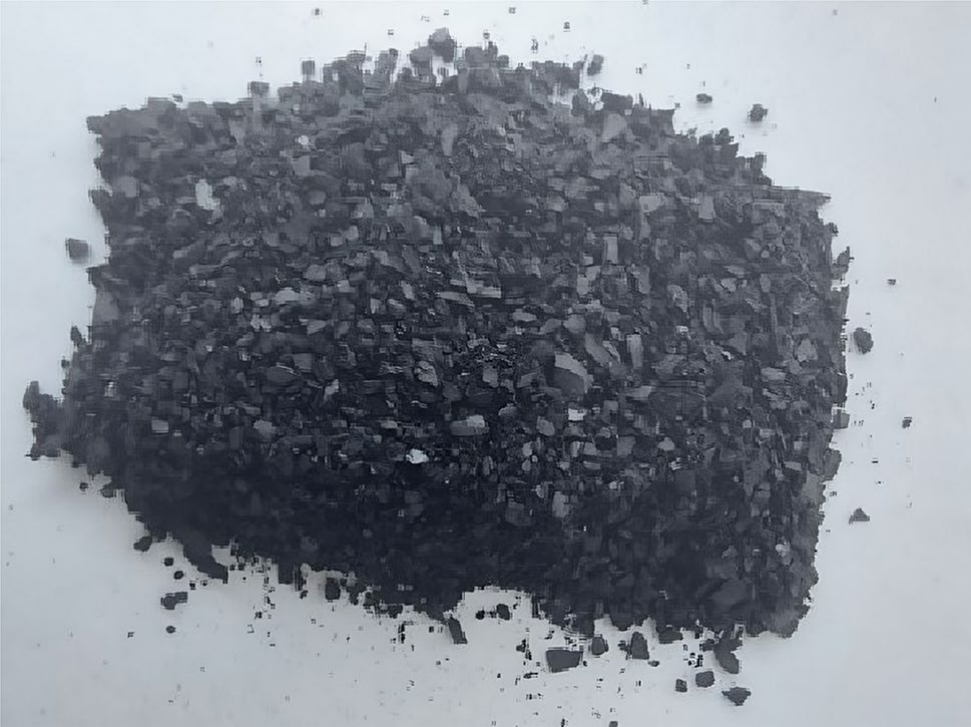
A close-up vision of melanin particles biosynthesized by *A. pullulans* AKW.

Following the preceding conditions, a mass production of melanin was conducted and subsequently isolated for the investigation of the chemical structure. The obtained melanin exhibits a black, granular appearance (Fig. 5). Generally, melanin is a heteropolymer consisting of diverse subunits, resulting in a complex, amorphous, and insoluble molecular structure. However, the specific composition of melanin varies depending on its source. Therefore, traditional methods face challenges when attempting to examine its constitution. Consequently, researchers employ several methods to explore melanin structure.

#### Scanning electron microscopy investigation

**Figure 6:**
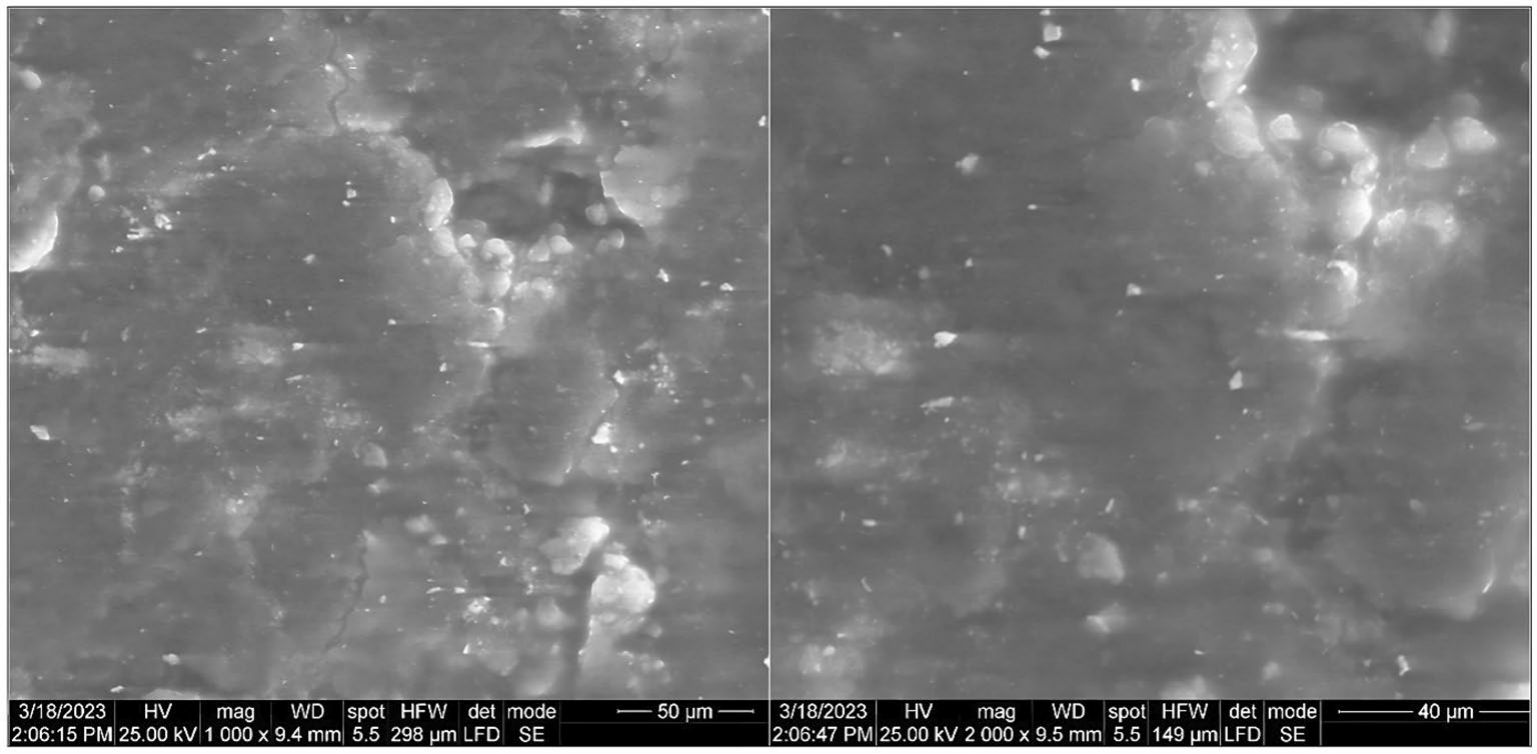
SEM micrographs of melanin pigment particles produced by *A. pullulans* AKW at the different indicated magnifications.

SEM analysis was applied to investigate the morphological features and architecture of melanin. The analysis provided a topography with high-magnification images of the surface of the melanin particles (Fig. 6). The hierarchical architecture of melanin particles produced by *A. pullulans* AKW was investigated by SEM. Generally, the particles of melanin polymer were shown in small compasses and aggregated.

#### X-ray diffraction pattern analysis

**Figure 7:**
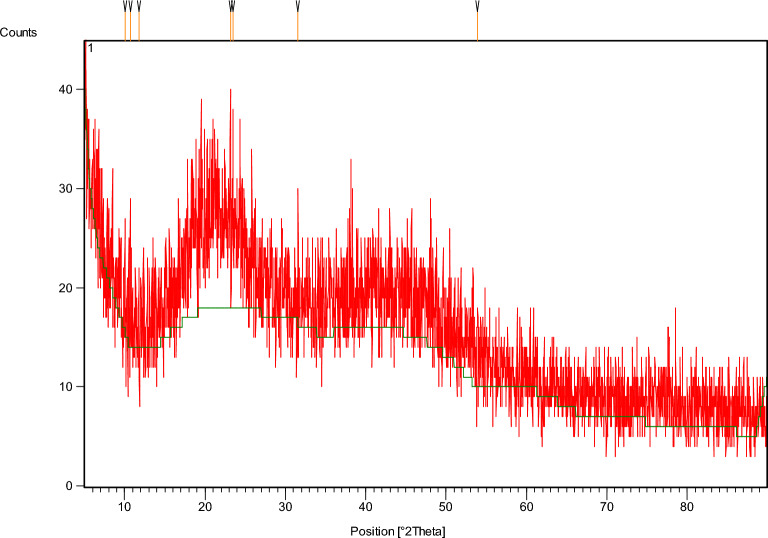
The XRD analysis of melanin.

The prepared melanin pigment was analyzed by XRD analysis to investigate its material type, phase, crystallographic properties, chemical composition, and physical features. The analysis depends on the constructive interference between the crystalline melanin and the monochromatic X-rays. The chart of XRD analysis is shown in Fig. 7, and the data is presented in Table 4.

**Table 4:** The XRD analysis data of melanin.

Pos. (°2Theta)	Height (cts)	FWHM (°2Theta)	*d*-spacing (Å)	Rel. int. (%)	Tip width (°2Theta)
10.0341	11.50	0.0787	8.81557	52.27	0.0945
10.6947	14.69	0.0590	8.27246	66.76	0.0708
11.7830	7.72	0.0984	7.51074	35.09	0.1181
23.1501	22.00	0.0787	3.84216	100.00	0.0945
23.4513	11.89	0.1968	3.79350	54.02	0.2362
31.5697	13.60	0.0394	2.83406	61.82	0.0472
53.8896	7.41	0.0960	1.69994	33.66	0.1152

#### Energy-dispersive X-ray spectroscopy

**Figure 8:**
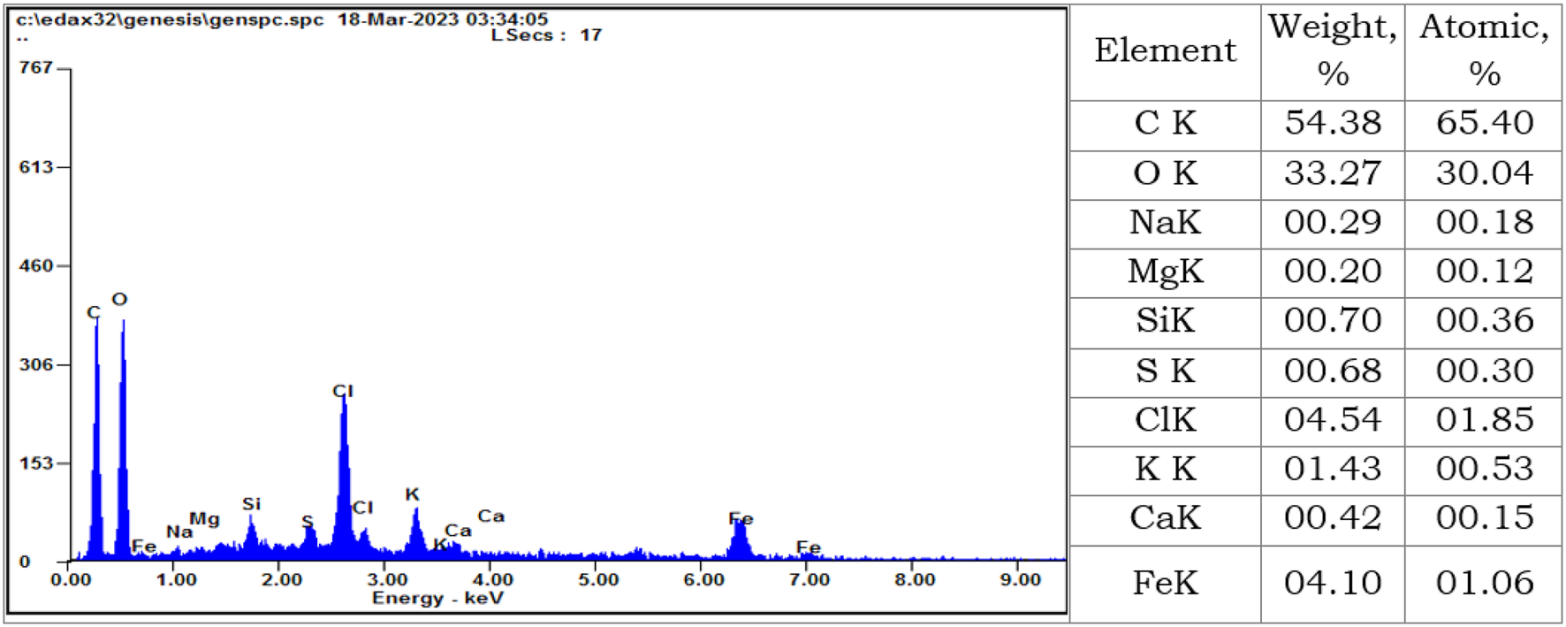
The EDX analysis of the purified melanin pigment.

The EDX analysis as an analytical technique was implemented for melanin pigment as shown in Fig. 8. The EDX analysis was applied to estimate the elemental analysis and chemical composition of the purified melanin pigment.

#### Thermogravimetric analysis

**Figure 9:**
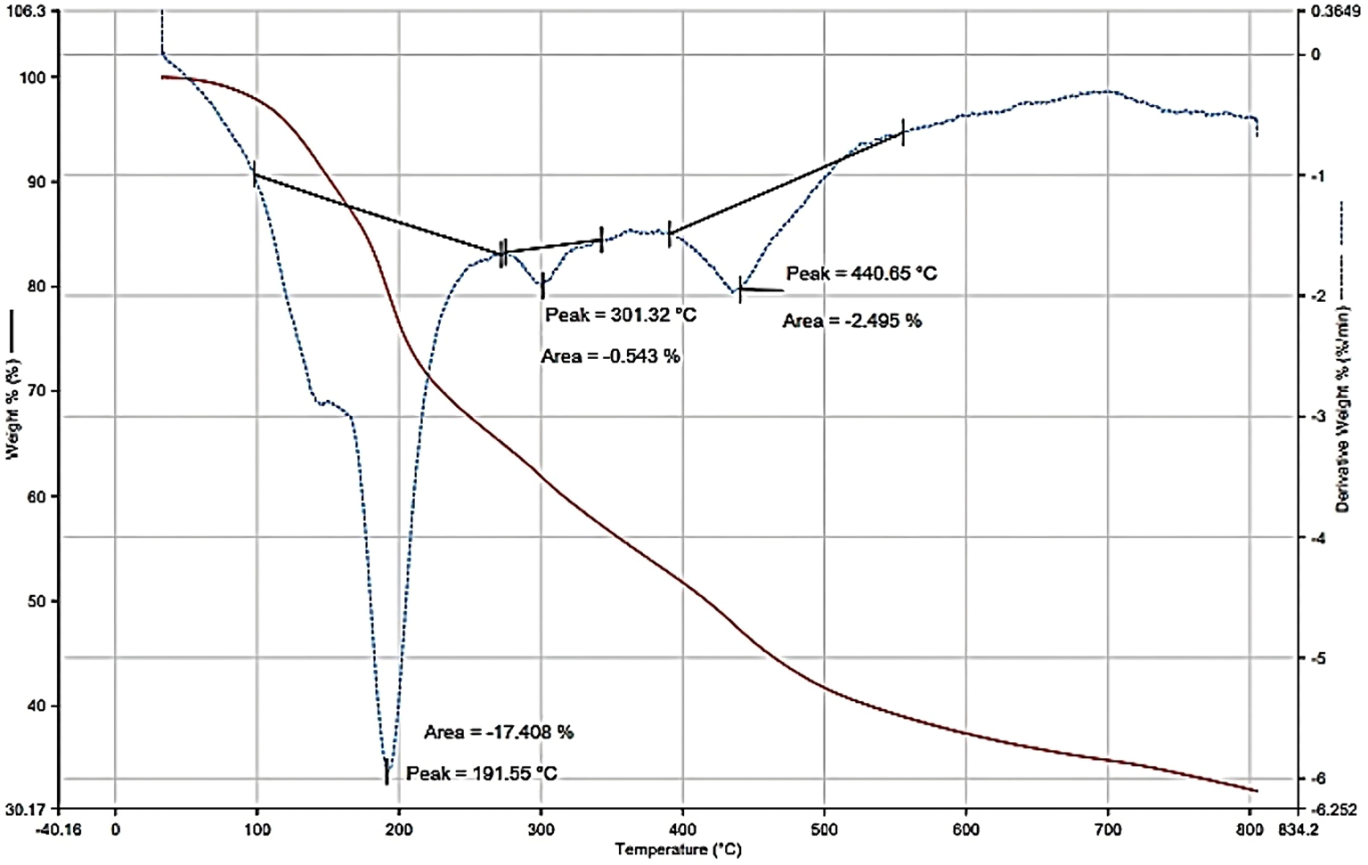
Thermogravimetric analysis of melanin.

The TGA was applied to investigate the thermal properties of the purified melanin pigment. Thus, the results as shown in Fig. 9 demonstrated the initial loss in the mass with a peak at 191.55 °C with indication of no residual adsorbed water.

## Discussion

The microbial fermentation process for optimizing melanin production is an auspicious procedure since microbial melanin is eco-friendly, biocompatible, and economical. Additionally, melanin polymer has a broad spectrum of applications, such as bioactive molecule (antioxidant, sunscreen, and antimicrobial agent), and could also be used in functional biomaterials as a sensitizer in dye-sensitized solar cells, and frame polymer for metal nanoparticles, as well as in bioremediation of metal-pollutant sites and extracellular electronic materials in microbial fuel cells^3,35^. Thus, the current study has been organized to maximize melanin production by the endophytic; *A. pullulans* AKW, using BBD, while ANN paradigm was applied for the first time.

Concerning the BBD paradigm, the center (middle) points of the tested factors achieved the maximum melanin production, being 10.811 ± 0.539 g/L (run 14). These results indicate that the selected level of each factor was accurate enough. As evidenced by the high F-values and low P-values in ANOVA, most of the model terms demonstrated a significant effect and lack of significant model error in comparison to the pure error, confirming the accuracy of the obtained data and the experimental design. Another, when the model has a high F-value and a non-significant lack of fit, it is indicative of adequate fitness. Contrary to tyrosine, incubation time and sucrose level attain a significant trend.

Commonly, the P-value is employed to detect the significance of the coefficients of various model terms. The significance level at the alpha ≤ 0.05 threshold signifies the incidence of a strong association between the tested variables and the response (melanin production)^25^.

The F-test hypothesizes no variations (null hypothesis, H_0_) between the model terms on the response. Instead, rejected H_0_ indicates the model terms are varied from each other (alternative hypothesis, H_1_). A large P-value (> 0.05) provides strong evidence to accept H_0_.^13,36^. Based on ANOVA, the H_0_ was rejected meaning the significance of the tested factors and reliability of the model in predicting melanin production by *A. pullulans* AKW within the investigated space.

Furthermore, for the model to be reliable, R^2^, adjusted R^2^, and pridected-R^2^ must be ≥ 0.75 and relatively near to each other^36^. The high R^2^ (0.9871), adjusted-R^2^ (0.9639), and pridected-R^2^ (0.9382) imply a good regression model, further, the relationship between the three tested variables and the melanin production is tight. A much lower predicted R^2^ than R^2^ is a warning sign that the model is overfitting the data, this is not applied to our case, where they are very close to each other^25^.

Based on the estimated coefficients the optimum level of the three variables, and the melanin response were predicted, which was very close to the actual melanin production, suggesting additional evidence for the accuracy of the model, and the design space, as well. The negative coefficient value indicates an antagonistic relationship between melanin production and the variable concentration in the tested design space, and vice versa. However, several studies reported BBD as an operative in signifying the factors reliable for melanin maximization, for instance, the optimization of the medium composition enhanced the production of melanin by *Aspergillus fumigatus*^12^.

The optimal ANN topology structure was denoted as 3–4–4–1, consisting of three input neurons, one output neuron, and two hidden layers of 4 neurons each, employing the activation function of NTanH(4)NTanH2(4).

Although artificial intelligence has become increasingly prevalent in recent scientific research, there has been no prior work on the modulation of microbial melanin production using ANN. This study supports this type of modeling.

ANN paradigm is elastic, able to predict well, and capable to learn any nonlinear function. Therefore, ANN can generate an efficient model from any type of data. The ANN platform utilizes a machine learning algorithm together with a flexible function for the input variable(s) for building a fully connected multilayer perceptron. This architecture uses indirect intermediate layers to predict the target melanin production by *A. pullulans* AKW. This modeling approach is more suitable when it is not necessary to elucidate the correlation between the response, and the inputs^22–24^.

Comparing the predicted vis residual values of both BBD and ANN models showed an even distribution along the 0-axis sides. A tight cluster of points was noted around the diagonal line. This distribution pattern confirms the high precision and adequacy of both models. Moreover, the residuals and the linear regression analysis of the ANN declared that both predicted and actual points were located very close to the line of ideal prediction than those of the BBD model, designating a well-trained ANN model, with better generality and higher precision.

The oval-shaped curve of the 3D plot for both BBD, and ANN indicates a strong and clear interaction between every pair of the tested factors, suggesting a careful choice of the factors, and the model fits the design well^9^.

However, contrarily to ANN, the prototype of ANN was not identical to BBD because ANN can discover complex nonlinear relationships between the inputs (tyrosine, sucrose, and incubation period) and the melanin production. ANN can identify concealed connections between input and output variables, even when they don't seem to have an explicit correlation. This is due to the two intermediate layers in ANN that manage unique correlation between inputs and output, rather than finding a direct route like observed in BBD. Thus, ANN is an excellent forecaster when the relationships between the output and input are not needed or identifiable^22–25^.

The performance of both models was evaluated by measuring RASE and AAE. ANN showed lower values for both errors’ measurements. Although the variation between the statistics of each model was small, the ANN model has a better generalization capability than BBD in melanin production. Experimental validation of the theoretical response of melanin production that was calculated by both models indicates the validity of both models.

ANN can predict with high accuracy because it can handle the system's nonlinearity better than other models that need only one-step calculation to approach the target^22,23^. The comparison of models from ANN and BBD requires some consideration of the differences between these two approaches. ANN is a type of machine learning model that mimics the behavior of the human brain, while BBD is a statistical approach used to optimize response variables based on experimental designs. Therefore, to compare models from ANN and BBD, it is required to clearly define the prediction accuracy, model interpretability, computational complexity, or ease of implementation. ANN models are often considered black-box models, while BBD models are typically more interpretable. Moreover, the modeling technique of ANN involves the building of network architecture, instituting the hidden layers and node numbers in each layer, training, and lastly, justification and authenticating the obtained model. Thus, the ANN paradigm is putatively more accurate because it encompasses all data points of experiments^22–25^. Furthermore, the robustness and generalization performance of ANN, especially on the new data, is better than the BBD model. Indeed, the ANN-robustness to noise or outliers surpassed BBD^22,23^. Therefore, the decision on the optimum conditions for melanin production was made based on the ANN model.

However, ANN has some limitations, such as taking longer computational time, for training and prediction, due to many iterations, and not showing the importance and influence of each factor in the model, which makes it hard to remove or simplify any factors from the model^22,32^.

Commonly, the regulation of melanin production depends on certain enzymes, i.e. tyrosinase with essentiality of sulfhydryl and lysyl residues in the enzyme active site^5^. Other nutritional conditions, e.g. glucose, peptone, and yeast extract are widely utilized in the nutritional medium. Moreover, numerous recent studies aimed to exploit agricultural residues for melanin productivity, such as corn steep liquor, fruit waste extract, and wheat bran, to minimize the cost, with a high yielding of melanin^37^. Additionally, there are several studies attaining optimization of fungal melanin, for example, Ribera et al.^38^ optimized melanin synthesis by *Armillaria borealis* with a yield of 11.58 g/L in a medium containing glucose, yeast extract, and tyrosine, over 97 days. Another study optimized melanin synthesis by the fungus, *Aspergillus fumigatus* (0.1 g/L) in a medium containing dextrose and peptone without tyrosine after 10 days^12^. The optimized melanin has been reported by *Auricularia auricula* (2.97 g/L), in a medium containing lactose, yeast extract, and tyrosine after 8 days^39^. Whereas, *Gliocephalotrichum simplex* produced melanin at 6.6 g/L after 6 days in a medium containing peptone and yeast extract^40^. However, in Table 5, the productivity of organisms in previous studies was calculated as g/day and compared with the current melanin yield by *A. pullulans* AKW was stated.

**Table 5:** Productivity of melanin by microorganisms including *A. pullulans* AKW relative to other previous studies.

Organism	Yield (g/L)	Incubation period (h)	Productivity (g/day)	References
Fungi				
*Aureobasidium pullulans* AKW	10.19	168	1.46	Current study
*Gliocephalotrichum simplex*	6.60	144	1.10	Jalmi et al.^40^
*Aspergillus fumigatus*	0.10	240	0.01	Raman et al.^12^
*Auricularia auricula*	2.97	192	0.37	Sun et al.^39^
*Armillaria borealis*	11.58	2328	0.12	Ribera et al.^38^
*Armillaria cepistipes*	27.98	3864	0.17	
*Daldinia concentrica*	1.78	1752	0.02	
Bacteria				
*Zymomonas mobilis*	3.20	120	0.64	Chávez-Béjar et al.^41^
*Nocardiopsis alba* MSA10	3.40	168	0.49	Kiran et al.^42^
*Bacillus safensis*	6.90	24	6.90	Tarangini and Mishra^43^
*Pseudomonas stutzeri*	6.70	10	16.08	Choi^8^
Actinomycetes				
*Streptomyces kathirae* SC-1	13.70	120	2.74	Guo et al.^44^
*Streptomyces* sp. ZL-24	4.20	120	0.84	Wang et al.^45^
*Streptomyces puniceus* RHPR9	0.39	168	0.06	Polapally et al.^46^

In comparison to these previous studies, based on the ANN paradigm, our strain (*A. pullulans* AKW) produced an appreciated amount of melanin (10.192 g/L) after a shorter incubation period (168 h) on a simple potato infusion medium, containing tyrosine (0.329%), and sucrose (4.870%). The biotechnological potential of the current strain in melanin production may be attributed to its constitutive production by the fungus that emerged as an endophytic fungus.

The endophytic microbiome, also known as endosymbiont, refers to a wide range of bacteria and/or fungi that live and develop inside or between cells of all types of plants without causing any harm to the host plant during at least some part of their life cycle. Endophytes are a newly emerged area of interest owing to their unique features^9^.

As revealed by BBD, the current *A. pullulans* AKW is tyrosine independent for melanin production. Following our results, *Pseudomonas stutzeri* produced melanin (6.7 g/L) within 10 h of incubation without tyrosine supplementation^8^. Contrarily, Surwase et al.^47^ found that L-tyrosine at 1.872 g/L enhanced melanin production by *Brevundimonas* sp SGJ, being 6.811 g/L. Generally, the metabolic pathways of melanin are depending upon the tyrosinase enzyme, which is a copper-dependent biocatalyst involved in ortho-specific hydroxylation and subsequent oxidation of monophenols like tyrosine^8^, in addition to the laccase enzyme which catalyzes the oxidation of a broad range of substrates like tyrosine including dihydroxy phenols and quinones^48^. Additionally, the mechanism of melanin could be conducted throughout different pathways, depending upon the random polymerization of a few building blocks e.g., L-tyrosine metabolites of indole-5,6-quinone, 5,6-dihydroxyindole carboxylic acid, 5,6-dihydroxyquinone carboxylic acid, dopamine, homogentisate, and some phenolic precursors^49^.

In general, two pathways are suggested for the production of microbial melanin, the first is the DOPA pathway which is catalyzed by tyrosinase and laccase, in which tyrosine is converted to L-DOPA, then to dopaquinone. The latter is extremely active and spontaneously oxidized and auto-polymerized to create melanin^3^. In the second DHN-pathway, malonyl-CoA is generated endogenously, then catalyzed by polyketide synthases, the sequential decarboxylative condensation of five malonyl-CoA molecules generates 1,3,6,8-tetrahydroxynaphthalene, which is subjected to cycles of reduction and dehydration reaction, generating 1,8-dihydroxynaphthalene (DHN), that polymerized finally to DHN melanin^3^. Another hypothesis for melanin synthesis in microorganisms suggested the formation of hydroxylated aromatic molecules, during the catabolic metabolism, e.g., such as homogentisic acid that accumulates due to enzymatic imbalance or interruption, this process generates other various sorts of melanin^50^. On this base, our data suggest that the current melanin of *A. pullulans* AKW is biosynthesized independently from tyrosine.

It is already known that the structure of melanin is complicated, in which it has various forms depending upon the polymerization pathway, building blocks, and enzymes, melanin is classified into several groups, including eumelanin, pyomelanin, pheomelanin, neuromelanin and allomelanin^8^. Therefore, the current melanin characteristics were elucidated.

The SEM investigation of melanin particle architecture confirmed the particles in small compasses in a definite crystal shape. Herein, the extracted pigmented melanin particles with average size lie between 150 and 160 nm. Commonly, the graphic particles of melanin showed to have a definite crystal shape, besides the particles shown in small compasses.

The XRD spectrum of the produced melanin sample is characterized by a broad peak, which is frequently perceived in amorphous and disordered materials balanced at about 23.1501. The Bragg reflection peak 2θ values were found for seven diffraction peaks at 10.0341, 10.6947, 11.7830, 23.1501, 23.4513, 31.5697, and 53.8896 consistent with tip width (°2Theta) 0.0945, 0.0708, 0.1181, 0.0945, 0.2362, 0.0472, and 0.1152, respectively. This peak is recorded owing to X-ray diffraction from parallel planer layers.

The Bragg equation was applied to calculate the inter-layer spacing *d*. The calculated values of *d* were found at 8.81557, 8.27246, 7.51074, 3.84216, 3.79350, 2.83406, and 1.69994 for the melanin sample. These findings are matched with the literature value of the inter-layer spacing in the stacked sheets model of melanin. The quality of the purified melanin was indicated by the proximity of the values of the grain size.

Moreover, the percentage of the crystallinity of melanin was calculated considering a glass substrate as a background. The melanin sample recorded the relative intensity percentages at 52.27, 66.76, 35.09, 100.0, 54.02, 61.82, and 33.66% for the seven diffraction peaks, respectively. These findings, regarding the lack of diffraction patterns related to the sample crystallinity, are in agreement with the results reported by Tarangini and Mishra^43^, and Xin et al.^51^. The crystallinity lack referred to the reliable physical features of melanin^52^.

The EDX analysis is particularly dependent on the interaction of X-ray excitation with the analyzed sample. The results indicated the amplest elements with the superlative compositions for carbon and oxygen atoms with weight of 54.38, and 33.27%, and atomic percent at of 65.40, and 30.04%, respectively. Our findings are in perfect conformity in all aspects with the results reported by Tarangini and Mishra^53^. The data also specified minor elements in the analysis with a total atomic % of 4.55%, this verified the purity of the produced melanin. The quantified minor elements were inferred as sodium/K (0.18%), magnesium/K (0.12%), silicon/K (0.36%), sulfur/K (0.30%), chlorine/K (1.85%), potassium/K (0.53%), calcium/K (0.15%), and iron/K (1.06%). The variation in the composition of melanin is supported by the factor of the change in the composition of the medium. Ribera et al.^38^ have supposed that infrequent elements such as sodium, potassium, and sulfur are interrelated to the growth medium composition. In addition, a considerable preliminary indication for the sample purity is the increased percentage of carbon besides the attendance of residual peptone in the raw melanin.

Likewise, the mass loss at 191.55 °C in the TGA analysis referred to the decomposition of peptone beside other residual growth media. Based on the literature reports^54^, the aliphatic chains or constituents decomposed below 400 °C, but the aromatic constituents started to decompose above 400 °C. regarding our melanin sample, the first decomposition occurred with the weight loss rate induced by 33% at 191.55 °C. The results in all characteristics indicated respectable thermal stability of melanin with a rate of weight loss of less than 20% unit 800 °C. These results regarding the intensive resistance of melanin to thermal degradation are in harmony with the previous literature^55^. It was worth mentioning that the raw melanin decomposed with loss of weight at 325 °C recording the highest loss in mass, while melanin was decomposed at 350 °C, these findings were formerly reported by Ribera et al.^38^ and approved with our results. Accordingly, the second loss in mass was recorded at 301.32 °C with a loss in mass lower than 80%, and the third loss in mass was recorded at 440.65 °C without significant loss in mass. Consequently, the residual-mass quantity for the solid sample of melanin did not show substantial variance (78%).

## Conclusion

To sum up, the optimization process of melanin was conducted by BBD and ANN models. l-tyrosine, sucrose, and incubation interval were the independent variables encountered in the BBD matrix. Both models were efficient in the determination of the best conditions for maximum melanin production. However, the ANN paradigm showed a superiority in the optimization process, yielding 10.192 ± 0.782 g/L. ANN accurately predicted melanin production and showed more improvement in melanin production by about 9.7% higher than BBD. Interestingly, L-tyrosine has no significant effect during the optimization process of melanin. The structure features of the produced melanin were established by SEM, XRD, EDX, and TGA analysis. SEM analysis depicted the aggregation of the melanin particles in small compasses. The XRD analysis specified the inter-layer spacing in the stacked sheets, and the crystallinity lack is referred to as  a reliable physical feature. The EDX analysis identified the maximal atomic % for carbon and oxygen atoms at 65.40, and 30.04%, respectively. The peptone decomposition in the TGA was recorded with the weight loss rate induced by 33% at 191.55 °C. Importantly, our results are thus the first to optimize melanin production by artificial intelligence paradigm on a simple medium using the new endophytic *A pullulans* AKW. The current work suggests developing new methods for the production of melanin by *A. pullulans* AKW on a higher scale, thus allowing its involvement in different products. Furthermore, a research paper could be performed to focus on the current state of the genetics behind microbial melanin production, as well as the potential for genetic engineering to improve microbial melanin production (Supplementary Information S1 and S2).

### Supplementary Information

Supplementary Information 1.

Supplementary Information 2.

## Data Availability

All data generated or analyzed during this study are included in this published article and the supplementary files.
