# Supplementary material for: A comparative study using response surface methodology and artificial neural network towards optimized production of melanin by Aureobasidium pullulans AKW
Source: Sci Rep. 2023 Aug 19;13:13545. doi: 10.1038/s41598-023-40549-z (PMC10439932; doi:10.1038/s41598-023-40549-z)
Supplement: Supplementary file 2 — Supplementary Information 2. [file 41598_2023_40549_MOESM2_ESM.docx]

**Fantastic report on**

**Document1**

**Distribution:**

| **Name** | **Function** | **0.1** | **0.5** | **1.0** |
| --- | --- | --- | --- | --- |
| N.N.-999 | Masterking Group Manager (MGM) | ✓ |  |  |
| N.N.-998 | Product Ontwikkelt Manager (POM) | ✓ |  |  |
| N.N.-1 | (AS) |  |  | ✓ |
| N.N.-2 | (DS) |  |  | ✓ |
| N.N.-3 | (BS) |  |  | ✓ |
| T.P. | Supervisor All Coding |  |  | ✓ |

#

# Table of Contents:

Table of Contents: 2

Anchor Scan Parameters: (Bookmark1) 3

Graphics: (Bookmark2) 3

Peak List: (Bookmark 3) 3

Identified Patterns List: (Bookmark4) 3

Plot of Identified Phases: (Bookmark 5) 3

Document History: (Bookmark 6) 3

# Anchor Scan Parameters: (Bookmark1)

Dataset Name 1

File name E:\X'Pert Data\2023\3\Dr Khaled xrd\1.xrdml

Comment Configuration=Stage Flat Samples, Owner=r, Creation date=2/28/2009 2:36:26 PM

Goniometer=PW3050/60 (Theta/Theta); Minimum step size 2Theta:0.001; Minimum step size Omega:0.001

Sample stage=PW3071/xx Bracket

Diffractometer system=XPERT-PRO

Measurement program=Program1, Owner=r, Creation date=7/7/2004 10:36:35 AM

Measurement Date / Time 3/18/2023 12:12:37 PM

Operator cwi

Raw Data Origin XRD measurement (*.XRDML)

Scan Axis Gonio

Start Position [°2Th.] 5.0100

End Position [°2Th.] 89.9900

Step Size [°2Th.] 0.0200

Scan Step Time [s] 1.0000

Scan Type Continuous

Offset [°2Th.] 0.0000

Divergence Slit Type Fixed

Divergence Slit Size [°] 0.4785

Specimen Length [mm] 10.00

Receiving Slit Size [mm] 0.1000

Measurement Temperature [°C] 25.00

Anode Material Cu

K-Alpha1 [Å] 1.54060

K-Alpha2 [Å] 1.54443

K-Beta [Å] 1.39225

K-A2 / K-A1 Ratio 0.50000

Generator Settings 30 mA, 40 kV

Diffractometer Type 0000000000005545

Diffractometer Number 0

Goniometer Radius [mm] 240.00

Dist. Focus-Diverg. Slit [mm] 91.00

Incident Beam Monochromator No

Spinning No

# Graphics: (Bookmark2)

# Peak List: (Bookmark 3)

| Pos. [°2Th.] | Height [cts] | FWHM [°2Th.] | d-spacing [Å] | Rel. Int. [%] | Tip width [°2Th.] | Matched by |
| --- | --- | --- | --- | --- | --- | --- |
| 10.0341 | 11.50 | 0.0787 | 8.81557 | 52.27 | 0.0945 |  |
| 10.6947 | 14.69 | 0.0590 | 8.27246 | 66.76 | 0.0708 |  |
| 11.7830 | 7.72 | 0.0984 | 7.51074 | 35.09 | 0.1181 |  |
| 23.1501 | 22.00 | 0.0787 | 3.84216 | 100.00 | 0.0945 |  |
| 23.4513 | 11.89 | 0.1968 | 3.79350 | 54.02 | 0.2362 |  |
| 31.5697 | 13.60 | 0.0394 | 2.83406 | 61.82 | 0.0472 |  |
| 53.8896 | 7.41 | 0.0960 | 1.69994 | 33.66 | 0.1152 |  |

# Identified Patterns List: (Bookmark4)

# Plot of Identified Phases: (Bookmark 5)

# Document History: (Bookmark 6)

Insert Measurement:

- File name = "1.xrdml"

- Modification time = "3/18/2023 1:32:24 PM"

- Modification editor = "cwi"

Default properties:

- Measurement step axis = "None"

- Internal wavelengths used from anode material: Copper (Cu)

- Original K-Alpha1 wavelength = "1.54060"

- Used K-Alpha1 wavelength = "1.54060"

- Original K-Alpha2 wavelength = "1.54443"

- Used K-Alpha2 wavelength = "1.54443"

- Original K-Beta wavelength = "1.39225"

- Used K-Beta wavelength = "1.39225"

- Incident beam monochromator = "No"

- Dist. focus to div. slit = "91.00000"

- Irradiated length = "10.00000"

- Spinner used = "No"

- Receiving slit size = "0.10000"

- Linear detector mode = "None"

- Length linear detector = "2"

- Step axis value = "0.00000"

- Offset = "0.00000"

- Sample length = "10.00000"

- Modification time = "3/18/2023 1:32:24 PM"

- Modification editor = "cwi"

Determine Background:

- Correction method = "Automatic"

- Bending factor = "21"

- Use smoothed input data = "Yes"

- Granularity = "18"

- Add to net scan = "Nothing"

- Modification time = "9/26/2022 9:58:01 AM"

- Modification editor = "cwi"

Determine Background:

- Correction method = "Automatic"

- Bending factor = "5"

- Use smoothed input data = "Yes"

- Granularity = "20"

- Add to net scan = "Nothing"

- Modification time = "2/22/2001 10:17:43 AM"

- Modification editor = "PANalytical"

Search Peaks:

- Minimum significance = "2.00"

- Minimum tip width = "0.01"

- Maximum tip width = "1.00"

- Peak base width = "2.00"

- Method = "Minimum 2nd derivative"

- Modification time = "2/20/2001 11:55:18 AM"

- Modification editor = "PANalytical"

Search & Match:

- Data source = Profile

- Restriction = "None"

- Scoring schema = "Multi phase"

- Auto residue = "Yes"

- Match intensity = "Yes"

- Demote unmatched strong = "Yes"

- Allow pattern shift = "No"

- Two theta shift = "0"

- Identify = "Yes"

- Max. no. of accepted patterns = "5"

- Minimum score = "27"

- Search depth = "6"

- Min. new lines / total lines = "40"

- Minimum new lines = "3"

- Minimum scale factor = "0.06"

- Modification time = "2/16/2001 11:03:07 AM"

- Modification editor = "PANalytical"

Determine Background:

- Correction method = "Automatic"

- Bending factor = "5"

- Use smoothed input data = "Yes"

- Granularity = "20"

- Add to net scan = "Nothing"

- Modification time = "2/22/2001 10:17:43 AM"

- Modification editor = "PANalytical"

Search Peaks:

- Minimum significance = "2.00"

- Minimum tip width = "0.01"

- Maximum tip width = "1.00"

- Peak base width = "2.00"

- Method = "Minimum 2nd derivative"

- Modification time = "2/20/2001 11:55:18 AM"

- Modification editor = "PANalytical"

Search & Match:

- Data source = Profile

- Restriction = "Restriction set"

- Description = "Common phases subfile only"

- All of: elements = ""

- At least one of: elements = ""

- None of: elements = ""

- Maximum no. of elements = "105"

- Skip marked as deleted by ICDD = "No"

- Skip marked as deleted by a user = "No"

- Skip non ambient pressure = "No"

- Skip non ambient temperature = "No"

- Quality marks set = ""

- Subfiles = "Common Phase"

- Scoring schema = "Multi phase"

- Auto residue = "Yes"

- Match intensity = "Yes"

- Demote unmatched strong = "Yes"

- Allow pattern shift = "No"

- Two theta shift = "0"

- Identify = "Yes"

- Max. no. of accepted patterns = "5"

- Minimum score = "27"

- Search depth = "6"

- Min. new lines / total lines = "40"

- Minimum new lines = "3"

- Minimum scale factor = "0.06"

- Modification time = "2/16/2001 3:30:09 PM"

- Modification editor = "PANalytical"

Smooth:

- Type of smoothing = "Polynomial"

- Polynomial type = "Low pass"

- Convolution range = "5"

- Modification time = "3/18/2023 1:33:14 PM"

- Modification editor = "cwi"

Search Peaks:

- Minimum significance = "0.50"

- Minimum tip width = "0.01"

- Maximum tip width = "1.00"

- Peak base width = "2.00"

- Method = "Minimum 2nd derivative"

- Modification time = "3/18/2023 1:33:41 PM"

- Modification editor = "cwi"

Determine Background:

- Correction method = "Automatic"

- Bending factor = "5"

- Use smoothed input data = "Yes"

- Granularity = "20"

- Add to net scan = "Nothing"

- Modification time = "2/22/2001 10:17:43 AM"

- Modification editor = "PANalytical"

Search Peaks:

- Minimum significance = "2.00"

- Minimum tip width = "0.01"

- Maximum tip width = "1.00"

- Peak base width = "2.00"

- Method = "Minimum 2nd derivative"

- Modification time = "2/20/2001 11:55:18 AM"

- Modification editor = "PANalytical"

Search & Match:

- Data source = Profile

- Restriction = "None"

- Scoring schema = "Multi phase"

- Auto residue = "Yes"

- Match intensity = "Yes"

- Demote unmatched strong = "Yes"

- Allow pattern shift = "No"

- Two theta shift = "0"

- Identify = "Yes"

- Max. no. of accepted patterns = "5"

- Minimum score = "27"

- Search depth = "6"

- Min. new lines / total lines = "40"

- Minimum new lines = "3"

- Minimum scale factor = "0.06"

- Modification time = "2/16/2001 11:03:07 AM"

- Modification editor = "PANalytical"

Search & Match:

- Data source = Profile

- Restriction = "Restriction set"

- Description = "Minerals subfile only"

- All of: elements = ""

- At least one of: elements = ""

- None of: elements = ""

- Maximum no. of elements = "105"

- Skip marked as deleted by ICDD = "No"

- Skip marked as deleted by a user = "No"

- Skip non ambient pressure = "No"

- Skip non ambient temperature = "No"

- Quality marks set = ""

- Subfiles = "Mineral"

- Scoring schema = "Multi phase"

- Auto residue = "Yes"

- Match intensity = "Yes"

- Demote unmatched strong = "No"

- Allow pattern shift = "No"

- Two theta shift = "0"

- Identify = "No"

- Modification time = "1/11/2001 10:27:07 AM"

- Modification editor = "PANalytical"

Determine Background:

- Correction method = "Automatic"

- Bending factor = "5"

- Use smoothed input data = "Yes"

- Granularity = "20"

- Add to net scan = "Nothing"

- Modification time = "2/22/2001 10:17:43 AM"

- Modification editor = "PANalytical"

Search Peaks:

- Minimum significance = "2.00"

- Minimum tip width = "0.01"

- Maximum tip width = "1.00"

- Peak base width = "2.00"

- Method = "Minimum 2nd derivative"

- Modification time = "2/20/2001 11:55:18 AM"

- Modification editor = "PANalytical"

Search & Match:

- Data source = Profile

- Restriction = "None"

- Scoring schema = "Multi phase"

- Auto residue = "Yes"

- Match intensity = "Yes"

- Demote unmatched strong = "Yes"

- Allow pattern shift = "No"

- Two theta shift = "0"

- Identify = "Yes"

- Max. no. of accepted patterns = "5"

- Minimum score = "27"

- Search depth = "6"

- Min. new lines / total lines = "40"

- Minimum new lines = "3"

- Minimum scale factor = "0.06"

- Modification time = "2/16/2001 11:03:07 AM"

- Modification editor = "PANalytical"

Search Peaks:

- Minimum significance = "0.50"

- Minimum tip width = "0.01"

- Maximum tip width = "1.00"

- Peak base width = "2.00"

- Method = "Minimum 2nd derivative"

- Modification time = "3/18/2023 1:37:40 PM"

- Modification editor = "cwi"
